# Hand Dominance Influences Motor Recovery Trajectories Following Stroke: A Longitudinal Study

**DOI:** 10.3390/s26175599

**Published:** 2026-09-03

**Authors:** Jennifer Gutterman, Gerard Fluet, Qinyin Qiu, Jigna Patel, Holly Gorin, Kiran K. Karunakaran, Karen J. Nolan, Emma Kaplan, Ashley Mont, Alma Merians, Sergei Adamovich

**Affiliations:** 1 Department of Rehabilitation and Movement Sciences, School of Health Professions, Rutgers, The State University of New Jersey, Newark, NJ 07101, USA; fluetge@shp.rutgers.edu (G.F.); qiuqi@shp.rutgers.edu (Q.Q.); patel421@shp.rutgers.edu (J.P.); merians@shp.rutgers.edu (A.M.); 2 Kessler Foundation, West Orange, NJ 07052, USA; kkarunakaran@kesslerfoundation.org (K.K.K.); knolan@kesslerfoundation.org (K.J.N.); ekaplan@kesslerfoundation.org (E.K.); 3 School of Engineering, Rutgers, The State University of New Jersey, Piscataway, NJ 08854, USA; ajmont@soe.rutgers.edu; 4 Department of Biomedical Engineering, New Jersey Institute of Technology, Newark, NJ 07015, USA

**Keywords:** stroke, hand dominance, motor recovery, upper limb, kinematics

## Abstract

Stroke often causes persistent upper extremity (UE) motor deficits, affecting reaching and grasping and impacting daily activities. Recovery may depend on whether the dominant or non-dominant hand is affected, a distinction that remains understudied. This pilot study aimed to characterize longitudinal UE recovery post-stroke. Twenty participants post-stroke (ten with the dominant hand affected, ten with the non-dominant hand affected) performed reach-to-grasp-and-lift tasks (1-inch cube, 2.5-inch circular object), plus clinical assessments, five times over six months with the initial testing around 15 days post-stroke (mean = 14.9). Kinematic measurements included Time to Peak Velocity, Time After Peak Velocity, Reach Duration, Reaching Trajectory Smoothness, Path Linearity, and Grasp Duration. Piecewise linear mixed-effects models evaluated longitudinal trends. Clinical measures improved significantly within 45 days post-stroke, while most kinematics improved significantly in the first 30 days but plateaued thereafter. A secondary exploratory analysis examining effects of hand dominance on UE recovery showed significant clinical improvements in both groups, but significant kinematic improvements were only demonstrated in the affected dominant hand group. Findings suggest kinematic improvements may be driven by dominant hand impairment, possibly reflecting motor control differences or greater reliance on the dominant hand. These results highlight the need for targeted rehabilitation strategies tailored to the non-dominant hand’s functional role.

## 1. Introduction

Stroke often causes persistent upper extremity (UE) motor deficits, particularly with reaching and grasping [1]. Upper limb weakness significantly impacts an individual’s ability to perform daily activities and negatively affects their quality of life [1,2]. These upper extremity motor function deficits are reflected in increased movement errors [3], reduced movement efficiency [4,5], and/or less-straight/more-curved reach paths at preferred speeds [6]. Often, these impairments persist even in the chronic phase of a stroke, despite standard rehabilitation interventions [7]. It is reported that about 1/3 of patients post-stroke fail to achieve full recovery of upper extremity function [8]. This highlights the need to better characterize how motor function changes over time post-stroke and identify the mechanisms and factors that influence these changes.

Traditional clinical assessments such as the Box and Block Test (BBT), Upper Extremity Fugl-Meyer Assessment (UEFMA), and the Action Research Arm Test (ARAT) are widely used to evaluate motor recovery and capacity in individuals with stroke [1,8]. However, clinical measures often lack the sensitivity needed to detect subtle deficits in movement quality and motor control [3,4]. As a result, commonly used clinical assessments can provide uncertain measures of a patient’s progress and improvement due to their limited sensitivity and potential floor and ceiling effects [9]. Moreover, clinical scores may continue to improve over time even when underlying movement quality has plateaued [10], suggesting that changes in performance can reflect altered movement strategies rather than true motor recovery [11]. Importantly, what is not captured in these clinical assessments are the different movement patterns that may be used to complete a movement task assessed by current clinical assessments [3]. This limitation of clinical measures has been increasingly recognized, as is the need for more ecological, valid measures to capture upper limb recovery following stroke, (e.g., [3,12]).

Kinematic analysis, which quantifies movement characteristics, can examine not just whether individuals with stroke can complete a movement but also how they perform that movement [3,13]. During recovery from stroke, one can use different movement patterns or different effectors to complete the same task [14,15]. Kinematic analysis provides a more sensitive and objective approach to detect nuanced changes in motor control [3,12,16], which is extremely valuable, especially during the critical early recovery period [13]. For example, studies using three-dimensional (3D) motion capture and other non-clinical assessments have shown that, even when clinical assessment indicates only mild impairment, individuals post-stroke exhibit less-efficient movements and higher movement variability than controls [3,6]. This affects the functional use of that limb and demonstrates that looking beyond clinical measures can help detect subtle movement impairments that might otherwise be overlooked.

Many studies have examined how the timing and dose of rehabilitation interventions influence upper limb recovery trajectories after stroke [8]. Recovery trajectories remain highly variable [8], and many individuals continue to experience persistent motor deficits months after their stroke [17,18]. To help explain these differences in recovery, studies have explored the role of other factors, such as initial impairment severity, hemisphere specialization, and lesion location [19,20,21,22,23].

However, recovery may also depend on whether the dominant or non-dominant hand is affected. This is an important distinction that remains largely understudied. For example, it has been shown that each hemisphere specializes in different aspects of motor control, which may influence pre-stroke differences in motor control and differing motor recovery patterns post-stroke [24,25]. Those with a left hemisphere stroke (thus, affecting the dominant right hand for right-handers) exhibit disruptions in the early phase of reaching, such as reduced smoothness [23]. In contrast, those with a right hemisphere stroke (affecting the non-dominant, left hand for right handers) often display disruptions in the later phases of the reaching movement [23]. However, most of this research has focused exclusively on right-handed individuals, making it difficult to disentangle the effects of hemisphere specialization from those of hand dominance [26].

Hand dominance is the preference to use one hand over the other when performing a task. The role of hand dominance in motor recovery remains understudied, despite its clinical importance [19,25]. Whether the dominant or non-dominant hand is affected may influence motor recovery, the development of targeted rehabilitation interventions [19,25], and/or the emergence of learned non-use [21]. While no baseline motor function differences have been shown between those with left and right hemisphere lesions (i.e., dominant versus non-dominant hand impairment in right-handed individuals) in chronic stroke patients, some evidence suggests that people with left hemisphere lesions, affecting the dominant hand in right-handers, show greater improvements in certain kinematic and strength measures following training [27]. One longitudinal study indicated that, although there were no differences in many clinical and functional measures between groups, the dominant hand group showed better recovery for fine dexterity, as shown through the Nine-Hole Peg Test, over the first three months post-stroke [22]. Given the potential for hand dominance to influence motor recovery, understanding its role could help guide the development of targeted interventions for individuals post-stroke [19,25]. Therefore, this study addresses this gap by longitudinally investigating how hand dominance affects upper extremity motor recovery trajectories following stroke.

In this pilot study, we used a longitudinal dataset from a larger clinical trial [28,29] that combines sensitive kinematic analysis with traditional clinical assessments to better understand upper limb recovery patterns. Uncovering these patterns can help inform the development of better targeted assessment and intervention strategies. The study combines 3D reach-to-grasp kinematics with clinical measures of body structure and function, as well as activity-level clinical measures, to provide a comprehensive picture of upper extremity recovery. The objectives of the study were (1) to characterize how upper extremity recovery post-stroke changes over 6 months post-stroke and, in a secondary exploratory analysis, (2) to examine whether trajectories differ depending on the dominance of the affected hand. We hypothesized that individuals post-stroke would continue to show improvements on clinical measures, while kinematic measures would plateau earlier, reflecting aspects of motor control not fully captured by clinical assessments. For the secondary analysis, we hypothesized that, while both groups would show similar early improvements on clinical measures, kinematic measures would reveal distinct motor recovery patterns related to whether the affected hemiplegic hand was the subject’s pre-morbid dominant or non-dominant hand. Specifically, we expected the affected dominant hand group to display a greater degree of change over time than the affected non-dominant hand group.

## 2. Materials and Methods

### 2.1. Participants

The 20 participants in this study were a subset of participants from an intervention study conducted at Kessler Foundation [29]. A total of 80 participants completed the original study from a Kessler Institute for Rehabilitation inpatient facility. Results from this parent study did not demonstrate any effects of dosage, timing, or type of therapy on the clinical outcomes [29]. Inclusion criteria for the intervention study were (a) a diagnosed stroke (ischemic or hemorrhagic) less than 30 days prior to the first reach-to-grasp data collection, (b) between 30 and 95 years of age, (c) able to follow instructions, (d) severe-to-moderate arm weakness (≥10/66 and ≤49/66 on the Upper Extremity Fugl-Meyer Assessment—UEFMA), and (e) intact cutaneous sensation. These criteria were selected to enroll participants in the early post-stroke phase with sufficient motor function to perform the task, while also minimizing ceiling effects on the UEFMA. Exclusion criteria included (a) upper extremity orthopedic pathology, (b) central nervous system pathology (MS, Parkinson’s, etc.).

**Figure 1:**
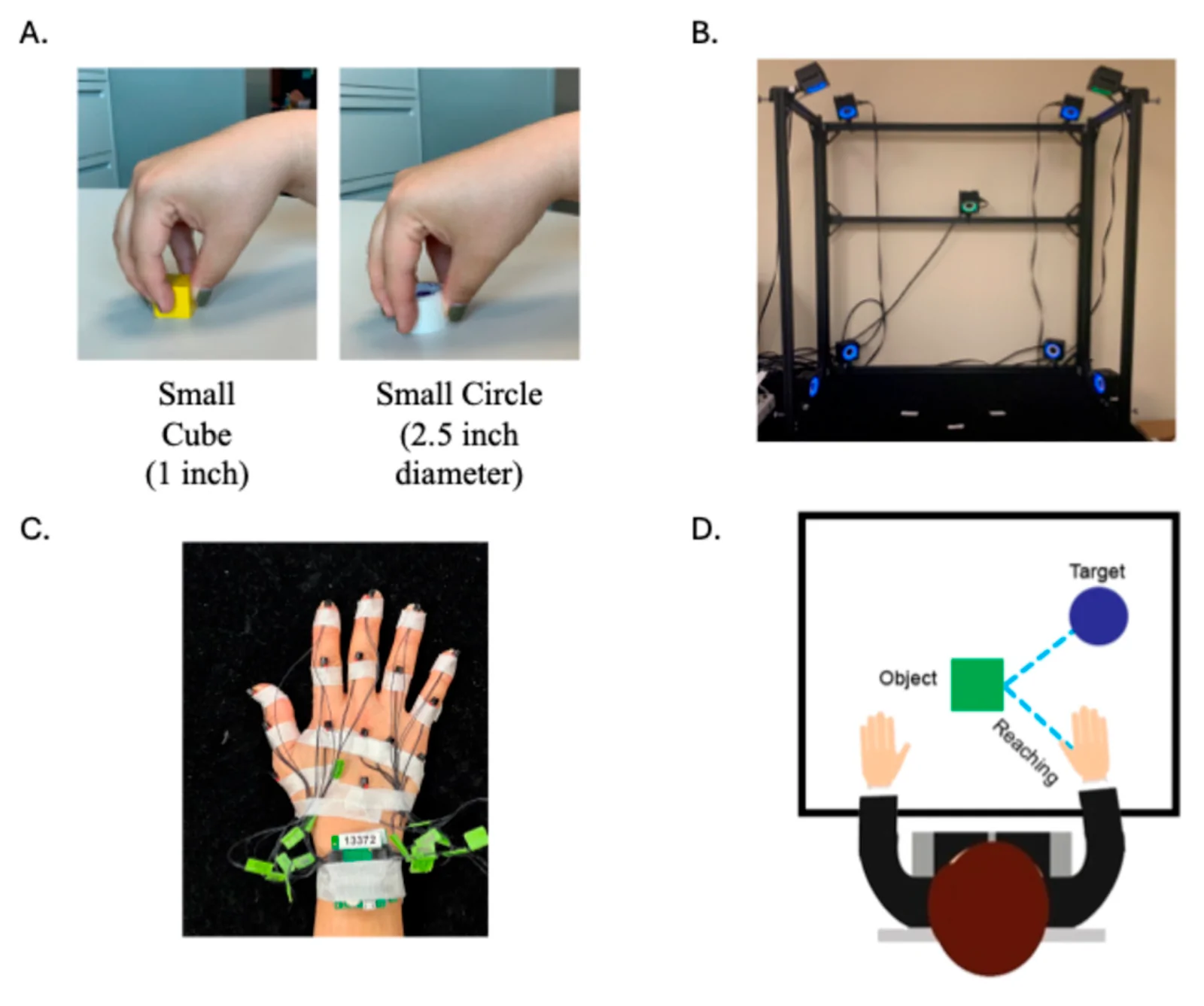
Experimental setup. (**A**) Objects used for the experiment. (**B**) Camera Setup. (**C**) Marker Placement. (**D**) Top view of the setup.

Inclusion criteria for this subset of participants were that they could grasp and lift two small objects, a 1-inch cube and a 2.5-inch circular object, with their affected arm at the first reach-to-grasp data collection session (see Figure 1A). In addition to the initial data collection, another criterion for this subset of participants was that they had to complete the final data collection as well. The participants performed 10 trials per object, for a total of 20 trials. Twenty participants (mean (SD) age of 59.05 (11.83)) were able to perform this during the baseline testing and were therefore included in the analysis (see Table 1 for participant information).

We split this subset into two groups: ten participants whose affected hand was their dominant hand (mean (SD) age of 54.4 (12.0) years; males = 6) and ten whose affected hand was their non-dominant hand (mean (SD) age of 63.7 (10.2) years, males = 5). We used these two groups (affected dominant hand and affected non-dominant hand) in the secondary analysis below.

### 2.2. Reach-to-Grasp Procedure

Reach-to-grasp (RTG) testing and clinical evaluation were conducted on the affected arm at five timepoints. The initial testing was performed 14.9 (6.6) days post-stroke (mean (SD)). The other four timepoints were during the second month post-stroke, one month after the previous testing, four months post-stroke, and six months post-stroke. RTG testing utilized an optical motion capture system where the cameras were positioned around an approximately 80 × 80 × 80 cm^3^ three-dimensional capture volume (Prime 13 OptiTrack cameras, Natural Point Inc., Corvallis, OR, USA) (see Figure 1B). Sixteen near-infrared active markers were placed on the dorsal surface of each fingertip, proximal interphalangeal, and metacarpophalangeal (MCP) joints plus an additional marker on the dorsal surface of the hand (see Figure 1C). The motion capture was performed using a custom-written script at a sampling frequency of 100 Hz. The signal from the OptiTrack was filtered using a low-pass second-order Butterworth filter, with a 10 Hz cutoff frequency (MATLAB v2025b). The marker positioned over the index finger’s MCP joint was used to calculate hand velocity and Reaching Trajectory Smoothness. Participants were positioned in a chair at a table, with their feet flat on the ground and their hips and knees in a 90-degree position. Their pronated right forearm and palm rested on a table, with the hand in front of them and 10 cm away from the acromioclavicular (AC) joint. Each object (a cube and a circular object) was positioned at midline, 15 cm from the participant’s chest. Objects were grasped and transported to a target 30 cm away from the participant’s torso, aligned with their AC joints. Participants were cued to (1) start reaching for the object at the command, “go”, (2) reach and pick up the objects with all five fingertips, (3) place it on the target area, and (4) return their hand to the starting position. Participants were encouraged to perform the activity as quickly as possible. The participants performed 10 trials for each object, for a total of 20 trials. The data were averaged across object types in this analysis. Figure 1D shows the experimental setup. This study focuses on the movements involved in reaching for and grasping objects; transporting the objects to the target and returning to the starting position were not analyzed. This paper limits its analysis to the reach and grasp phases of this activity because both proximal and distal effectors make substantial contributions to these phases.

### 2.3. Kinematic Measurements

**Figure 2:**
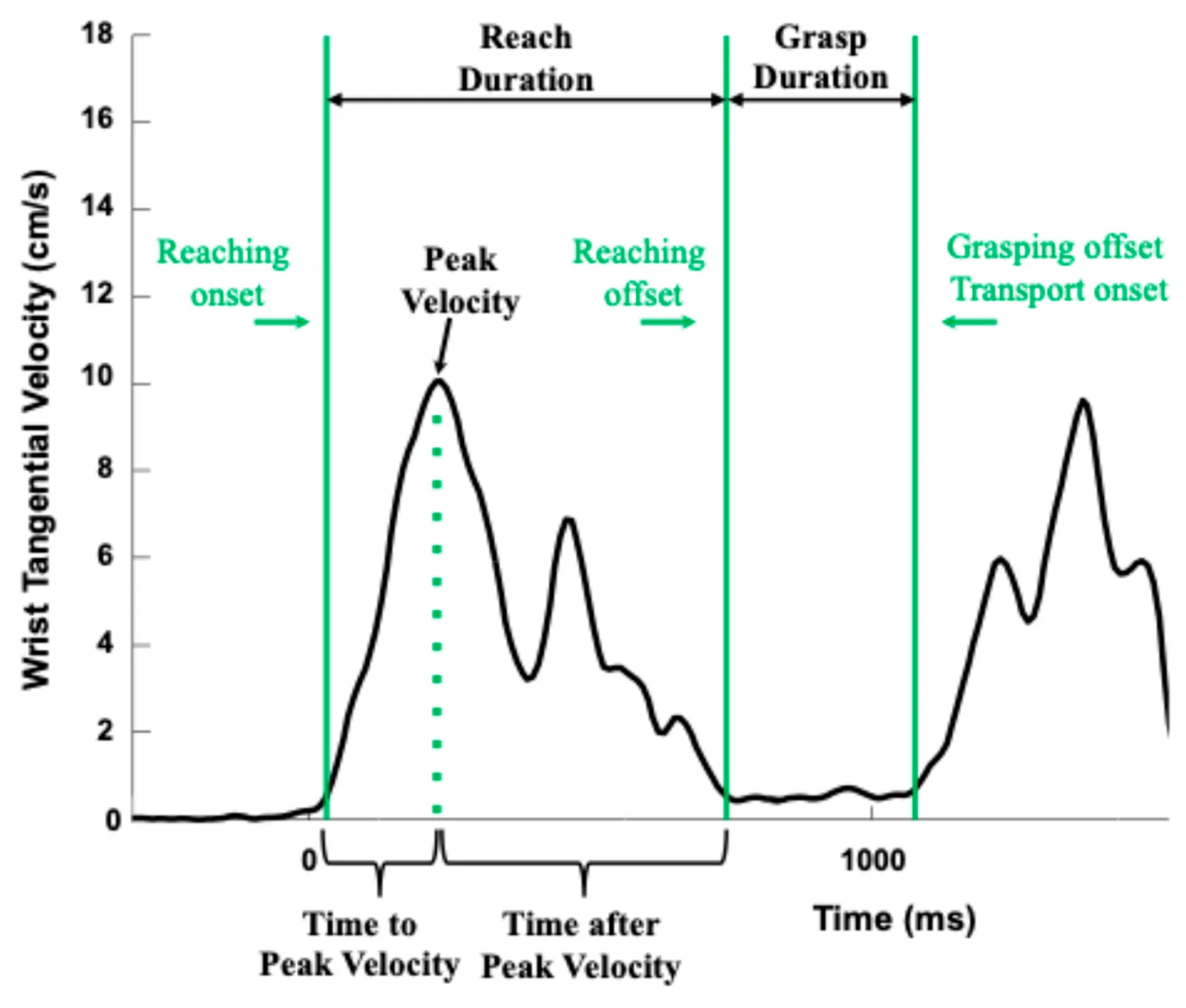
Example trace of wrist tangential velocity. The green vertical lines represent the timepoints during the reach-to-grasp variables we analyzed. The dotted vertical line represents peak velocity during Reach Duration. Time to Peak Velocity is represented by the first solid green vertical line to the green dotted vertical line. Time After Peak Velocity is represented by the green dotted vertical line to the second solid green vertical line. Grasping offset/transport onset indicates the onset of the transport of the grasped object to the target (see Figure 1B).

Figure 2 shows an illustrative hand tangential velocity profile, with annotations that demonstrate how the kinematic variables (e.g., peak velocity, onset and offset) were measured. Velocity was calculated as:

$$v(t)=\frac{dp(t)}{dt}$$

where **p**(*t*) = [*x*(*t*),y(*t*),*z*(*t*)], and *x*, *y*, and *z* are the marker’s three-dimensional coordinates. Peak velocity (PV) was defined as the maximum wrist tangential velocity measured during reach. The reaching movement’s onset was defined as the time at which the wrist velocity exceeded 5% of the PV achieved during the reaching movement. Since participants started each reaching movement with their arm resting on the table, the onsets of the affected arm’s reaching movements were reliably identified, despite the noisy velocity profile. The reaching offset was defined as the first point in time at which the wrist velocity fell below 5% of PV, consistent with prior kinematic studies of paretic upper limb movements [30]. When wrist velocity fell below 5% of the PV achieved during the reaching movement, the grasping offset (transport onset) was defined as the time at which wrist velocity subsequently exceeded 5% of the PV achieved during the transport phase. All velocity profiles were visually inspected, and onset and offset detections were corrected when necessary. If wrist velocity did not fall below 5% of PV, we selected the lowest velocity point as the reaching offset, and defined the grasp offset as the subsequent timepoint at which velocity continuously rose again.

The movement period between reaching onset and reaching offset was used to calculate Time to and After Peak Velocity, Reach Duration, Reaching Trajectory Smoothness of the hand spatial trajectory, and Path Linearity. Time to Peak Velocity (TTPV) was the time elapsed between the initiation of reaching and the time at which PV was reached. Time After Peak Velocity (TAPV) was the time period beginning at PV through the end of the reach. Reach Duration was calculated as the sum of TTPV and TAPV, representing the total time from reach onset to reach offset. Reach Duration reflects how quickly the participant moved their hand towards the object. The second derivative of hand tangential velocity v(t) produced tangential jerk. Reaching Trajectory Smoothness (RTS) was quantified by normalizing the integrated squared jerk by the trajectory length and movement duration:

$$\mathrm{Smoothness}=\sqrt{\frac{1}{2}\left(\int_{0}^{T}{j(t)}^{2}dt\right)\frac{T^{5}}{L^{2}}}$$

where j(t) is tangential jerk, T is movement duration, and L is the hand trajectory length. This normalized jerk-based metric quantifies movement smoothness, with lower values indicating smoother movement. RTS of the wrist spatial trajectory was measured by calculating log-transformed jerk during the reaching movement. Path Linearity was calculated as the ratio of the actual reaching trajectory and the linear distance from the wrist starting position to the wrist final position during reach. Grasp Duration was defined as the time from reaching offset (the end of the reach phase) to grasping offset/transport onset, when participants initiated transport of the object to the target (see Figure 2 for an illustration of the kinematic measures).

### 2.4. Clinical Measurements

The Box and Block Test (BBT) was conducted to assess gross manual dexterity [31]. The Upper Extremity Fugl-Meyer Assessment (UEFMA) was collected to describe motor recovery at the impairment level on a scale of 0–66 [32] and the Action Research Arm Test (ARAT) to describe motor function at the activity level on a scale of 0–57 [33]. These measurements were collected at each of the five data collection points.

### 2.5. Data Analysis

For both the primary and secondary analyses, a cubic smoothing spline was applied to the five original data collection timepoints to model recovery trajectories across key timepoints. The spline was used to generate estimated values at six timepoints fixed across all participants (days 15, 30, 45, 60, 120, and 180), with inclusion criteria requiring participants to have both the initial and final data collections. The spline was performed because of the variability in the number of days post-stroke at the time of the test, as data collection rarely occurs at precisely equally spaced points between tests [34]. For each outcome variable, individual longitudinal recovery trajectories were estimated using cubic smoothing splines (smooth.spline function in R) as a function of time since stroke. All available observations for each participant were used in the spline fitting (all.knots = TRUE), and a fixed smoothing parameter (spar = 0.1) was applied across participants to capture non-linear recovery patterns while also providing for smooth trajectory estimates. To preserve the observed baseline point of each recovery trajectory, the baseline measurement was assigned a large weight (w = 10^6^). This approach was used due to the clinical importance of a baseline assessment when examining recovery post-stroke. This method accounts for the variability in the timing of the baseline test due to scheduling issues as well as the rapid recovery immediately post-stroke. Additionally, three participants had one missing session (missing data = 3%), which was accommodated in the spline-based analysis.

Using the spline estimated values at the six fixed timepoints, a piecewise linear mixed-effects model was implemented to evaluate longitudinal trends, allowing for flexible estimation of phase-specific slopes (fixed effects), while accounting for day 15 differences between individuals through random intercepts [35]. This approach has been utilized to examine rates of change in functional measures across different phases post-stroke, including the post-stroke period of heightened neuroplasticity as well as long-term trajectories [36,37,38]. The assumptions of the piecewise linear mixed-effects models were examined by evaluating residuals for linearity, homoscedasticity and normality. Linearity and homoscedasticity were evaluated by visually inspecting plots of residuals versus fitted values. Normality of residuals was assessed using Q-Q plots and histograms. No substantial violations of model assumptions were observed. Influential data points were identified using Cook’s distance [39,40]. When influential points were detected, robust linear mixed-effects models were fit, which reduce the weight of influential data points by using the R package “robustlmm” [41]. This approach has been used in studies of motor recovery following stroke to retain all data, while limiting the influence of outliers [42]. Due to robust linear mixed-effects models not providing exact *p*-values, the reported *p*-values for fixed effects in the robust linear mixed-effects model were approximated by combining the Satterthwaite-estimated degrees of freedom from the standard mixed-effects model with the t-statistic from the robust model [43]. To account for multiple comparisons, we applied the Bonferroni–Holm correction to the target alpha rate of 0.05. All *p*-values are reported post-correction.

For the primary analysis (all 20 participants), longitudinal models were fit for each clinical and kinematic variable using the spline-estimated values for all 20 participants, to characterize overall upper extremity recovery trajectories over six months. An influential data point was detected for BBT, and therefore a robust linear mixed-effects model was applied as described above for this variable.

For the secondary analysis (dominant vs. non-dominant groups), day 15 values for all variables were compared between groups using a *t*-test to assess initial group differences. When the assumption of normality was not met, as determined by the Shapiro–Wilk Test, we used the Mann–Whitney U test for group comparisons at baseline. Influential data points were detected for BBT, TTPV and Grasp Duration for the affected dominant hand group. Therefore, analyses were performed for these variables using a robust linear mixed-effects model.

Spline interpolation, as well as testing of the normality assumptions for the piecewise linear models and the piecewise models, was performed in RStudio (Version 2024). Normality assumption testing and statistical tests for correlations and comparisons between groups were performed in SPSS (Version 29.0.2.0). All figures were done using RStudio.

## 3. Results

### 3.1. Clinical Recovery Patterns Post-Stroke

Piecewise linear mixed-effects models demonstrated significant improvements in clinical measures for the BBT, ARAT and UEFMA during the initial day 15 to day 30 interval (BBT: β = 0.62, *p* < 0.001; ARAT: β = 0.79, *p* < 0.001; FM: β = 0.78, *p* < 0.001), as well as the day 30 to day 45 interval for all three clinical measures (BBT: β = 0.54, *p* < 0.001; ARAT: β = 0.57, *p* < 0.001; UEFMA: β = 0.49, *p* < 0.001). After 45 days, recovery appeared to plateau, with no significant changes in subsequent time intervals for the ARAT or UEFMA. However, robust piecewise linear mixed-effects models demonstrated significant BBT score improvements during the day 45 to day 60 interval (β = 0.31, *p* = 0.03) and the day 60 to day 120 interval (β = 0.13, *p* < 0.001). For all clinical outcome results, see Table 2.

### 3.2. Kinematic Recovery Patterns Post-Stroke

**Figure 3:**
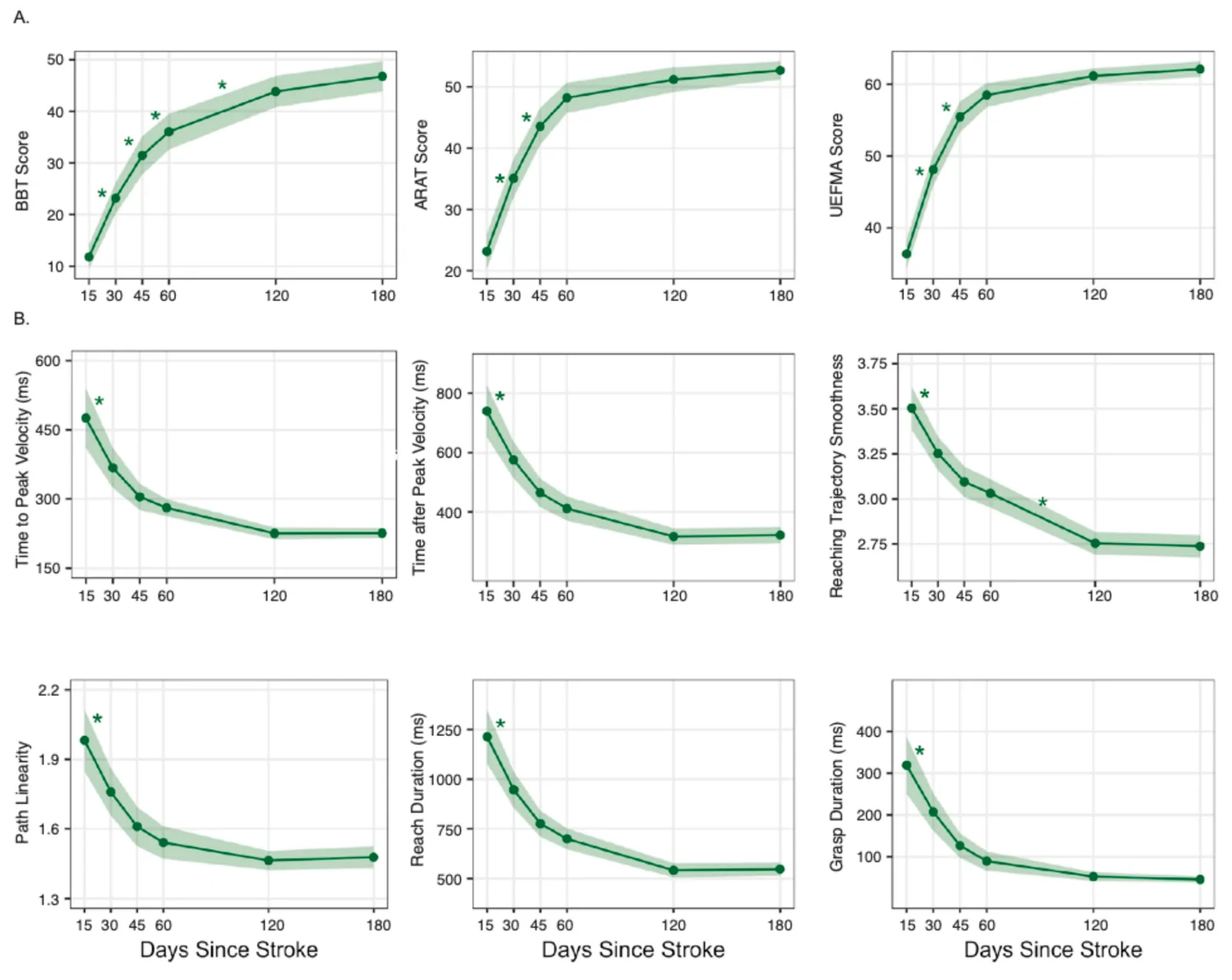
Piecewise linear mixed-effects models for clinical and kinematic variables. Group means are denoted by the corresponding dots with shaded areas indicating mean ± standard error (SE). (**A**) clinical variables and (**B**) kinematic variables. BBT = Box and Block Test; ARAT = Action Research Arm Test; UEFMA = Upper Extremity Fugl-Meyer Assessment. An asterisk (*) denotes a significant change between the two adjacent timepoints.

For all kinematic variables, piecewise linear mixed-effects models revealed significant improvements early in recovery, as there were significant improvements between day 15 and day 30 for all kinematic variables (TTPV: β = −7.20, *p* = 0.03; TAPV: β = −10.97, *p* = 0.01; RTS: β = −0.02, *p* < 0.01; Path Linearity: β = −0.01, *p* = 0.02; Reach Duration: β = −17.81, *p* < 0.01; Grasp Duration: β = −7.47, *p* = 0.04). TTPV, TAPV, Path Linearity, Reach Duration and Grasp Duration recovery all plateaued after day 30. While there was a plateau between days 30 and 45 and 45 to 60 for RTS, significant improvements were shown from day 60 to day 120 (β = −0.005, *p* < 0.01). For all kinematic outcomes, see Table 3 and Figure 3.

### 3.3. Secondary Analysis: Clinical and Kinematic Recovery by Hand Affected Post-Stroke

#### 3.3.1. Baseline Differences Between Groups

The *t*-tests and Mann–Whitney tests, when appropriate, showed no baseline differences between the affected dominant hand group and the affected non-dominant hand group at baseline (day 15) for any of the clinical or kinematic measures (all *p* > 0.05, see Table 4).

#### 3.3.2. Clinical Measure Recovery Patterns Post-Stroke

##### Affected Dominant Hand

For participants in the affected dominant hand group, piecewise linear mixed-effects models demonstrated significant improvements in clinical measures, including the BBT, ARAT and UEFMA during the initial 15 to 45 days post-stroke. After applying the Holm–Bonferroni correction to account for the multiple time interval comparisons for each clinical measure, significant improvements were observed during the initial day 15 to day 30 interval (BBT: β = 0.73, *p* = 0.001; ARAT: β = 0.93, *p* < 0.001; FM: β = 0.84, *p* < 0.001), as well as the day 30 to day 45 interval for all three clinical measures (BBT: β = 0.65, *p* = 0.004; ARAT: β = 0.59, *p* = 0.01; UEFMA: β = 0.51, *p* < 0.001). After 45 days, recovery appeared to plateau, with no significant changes in subsequent time intervals for any of the clinical measures. For all clinical outcome results, see Table 5.

##### Affected Non-Dominant Hand

Piecewise linear mixed-effects models also revealed significant improvements for the affected non-dominant hand group during the first 45 days post-stroke across all three clinical measures including the BBT (days 15–30: β = 0.54, *p* = 0.004; days 30–45: β = 0.45, *p* = 0.02), the ARAT (days 15–30: β = 0.66, *p* = 0.003; days 30–45: β = 0.45, *p* = 0.01), and the UEFMA (days 15–30: β = 0.72, *p* < 0.001; days 30–45: β = 0.47, *p* = 0.005). Significant improvements were also observed during the day 60 to day 120 time interval for the BBT (β = 0.15, *p* = 0.002). However, no significant improvements were demonstrated during the time intervals of day 45 to day 60 or day 120 to 180 post-stroke for BBT, or during the time intervals of days 45 to 60, 60 to 120, or 120 to 180 days post-stroke for ARAT or UEFMA.

#### 3.3.3. Kinematic Recovery Patterns Post-Stroke

##### Affected Dominant Hand

**Figure 4:**
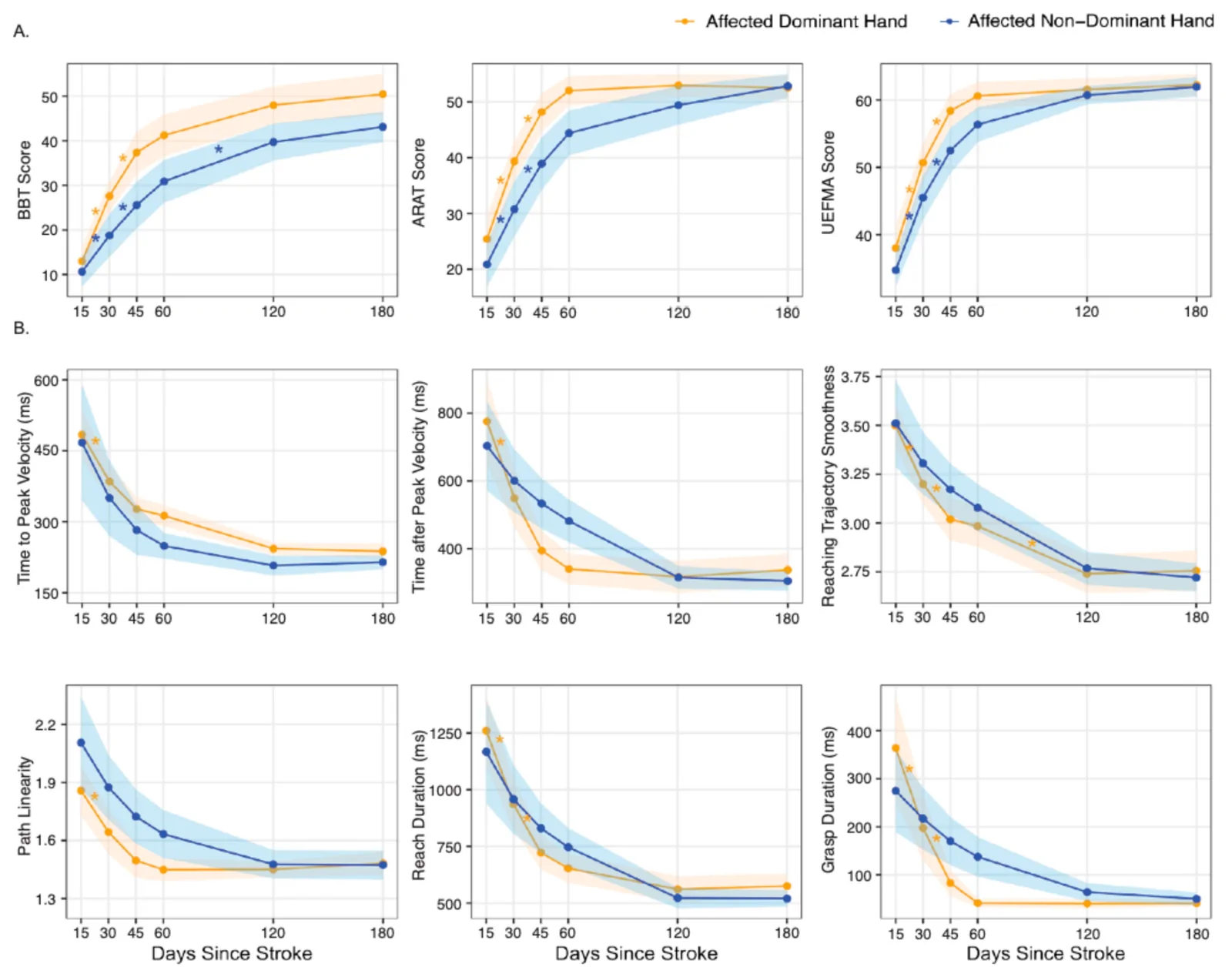
Piecewise linear mixed-effects models for clinical and kinematic variables by hand dominance. Group means are denoted by the corresponding dots with shaded areas indicating mean ± standard error (SE). (**A**) Clinical variables and (**B**) kinematic variables. BBT = Box and Block Test; ARAT = Action Research Arm Test; UEFMA = Upper Extremity Fugl-Meyer Assessment. An asterisk (*) denotes a significant change between the two adjacent timepoints.

Robust piecewise linear mixed-effects models demonstrated improvements in TTPV for the affected dominant hand group (shorter time) from day 15 to day 30 (β = −6.34, *p* = 0.006, see Figure 4 and Table 6). For the affected dominant hand group, the piecewise linear mixed-effects model showed TAPV significantly improved between day 15 to day 30 (β = −15.08, *p* = 0.007). The affected dominant hand group also demonstrated improvements in RTS from day 15 to day 30 (β = −0.02, *p* < 0.001), from day 30 to day 45 (β = −0.01, *p* = 0.03) and from day 60 to day 120 (β = −0.004, *p* < 0.001). Path Linearity significantly improved from day 15 to day 30 for the affected dominant hand group (β = −0.01, *p* = 0.02). In this group Reach Duration significantly improved from day 15 to day 30 (β = −21.65, *p* < 0.001) and from day 30 to day 45 (β = −14.22, *p* = 0.03). For Grasp Duration, a robust piecewise linear mixed-effects model demonstrated that the affected dominant hand group significantly improved (shorter time) between day 15 and day 30 (β = −8.70, *p* < 0.001) and between day 30 to day 45 (β = −4.74, *p* = 0.01; see Figure 4 and Table 6). However, after 30 days, recovery appeared to plateau, with no significant changes in subsequent time intervals for TTPV, TAPV or Path Linearity. No significant improvements were shown from day 45 to day 60, day 60 to day 120 or day 120 to day 180 for Reach Duration and Grasp Duration, and from day 45 to day 60 and day 120 to day 180 for RTS.

##### Affected Non-Dominant Hand

The affected non-dominant hand group did not show significant improvements for TTPV for any timepoint (all *p* > 0.05, see Figure 4 and Table 6). For TAPV there were no significant improvements shown for the affected non-dominant hand group (all *p* > 0.05). There were no significant improvements for the affected non-dominant hand group for RTS (all *p* > 0.05) or Path Linearity at any point as well (all *p* > 0.05). The affected non-dominant hand group did not significantly improve at any of the timepoints for Reach Duration (all *p* > 0.05), and no significant improvements were observed for Grasp Duration for this group (all *p* > 0.05). See Figure 4 and Table 6 for all kinematic measures for both groups.

## 4. Discussion

This study used longitudinal data to examine motor recovery trajectories over the course of six months post-stroke. In our study, the UEFMA and ARAT scores of the entire 20 subject cohort improved up to day 45, and the BBT scores continued to improve until day 120. In contrast, most of the kinematic measures for the twenty-subject cohort only showed significant improvements between day 15 and day 30. Reaching Trajectory Smoothness, however, demonstrated significant improvements both between days 15 and 30 and between days 60 and 120. This study used kinematic measures collected with an optical motion capture system, which utilizes an array of 16 active markers in individuals with acute stroke. This study demonstrated how sensor technology can be used in a rehabilitation hospital setting to collect kinematic data that may produce finer-grained analyses of motor control than traditional clinical measurements. For example, with the use of the sensory technology, this study suggests that recovery may be based on the role of the hand (i.e., whether the dominant versus non-dominant hand is affected). Overall, these results suggest that kinematic measures may reveal changes in movement quality patterns that are not apparent when analyzing clinical measures alone.

Our results are similar to those of Cortes et al., who found that UEFMA and ARAT scores continued to improve beyond the time when the kinematic variables had plateaued [10]. The discrepancy between the clinical and kinematic measures may reflect changes in movement strategies, including compensatory limb or trunk use, for example, rather than true motor recovery [11,12]. Previous work has suggested that kinematic measures provide for a more nuanced assessment of post-stroke upper extremity recovery [44]. In addition, Lang et al. observed improvements in reaching efficiency over the first 90 days post-stroke [4]. In our study, Path Linearity plateaued after day 30, whereas Reaching Trajectory Smoothness, an integrative measure of inter-joint control, improved between days 15 and 30 and again between days 60 to 120. This suggests that some integrative measures of movement can continue to improve after day 30, more closely mirroring the prolonged changes observed in clinical measures. Lang et al. also demonstrated significant improvements in reach movement time and reach-to-grasp movement time between the first week and 90 days post-stroke [4]. Wagner et al. similarly reported significant differences in reaching performance between the acute phase and subacute phases, with a mean assessment around 109 days post-stroke [45]. Although our study showed a plateau in most of the kinematic measures after day 30, these differences may partly reflect variation in sampling schedules. In particular, our multiple intermediate timepoints may have allowed us to detect narrower periods of change.

The secondary aim in the study was to examine how hand dominance is associated with motor recovery trajectories following stroke. Both groups showed significant improvements in clinical measures during the first 45 days post-stroke, with the affected non-dominant hand group also displaying significant BBT score improvements between day 60 and day 120. However, only the affected dominant hand group showed significant improvement in the kinematic variables, while the affected non-dominant hand group did not demonstrate statistically significant improvements in the kinematic variables at any timepoint. Thus, although kinematic measures improved in both groups when considered together, the findings suggest these effects appear to be primarily driven by participants with an affected dominant hand.

The affected dominant hand group demonstrated rapid improvements in several kinematic variables, followed by a plateau. This is consistent with previous studies that found early improvements in kinematic variables predominantly between 4 and 6 weeks post-stroke, followed by a plateau, (e.g., [10,30,46,47]). Specifically, we observed early improvements for the affected dominant hand in Time to Peak Velocity, Time After Peak Velocity, Reaching Trajectory Smoothness, Path Linearity, Reach Duration, and Grasp Duration. Similarly, Van Kordelaar et al. found that movement duration improved (shorter time) up until 5 weeks [30], which is similar to Reach Duration and Grasp Duration improving up until 45 days in our study. Additionally, Thrane et al. showed smoothness improvements up to around 4 weeks post-stroke, followed by a plateau [46]. Together, these improvements in these variables are consistent with previous studies showing that recovery of upper extremity performance is most pronounced during the first weeks to months following stroke, a period associated with heightened neuroplasticity [30].

The finding that kinematic improvements were demonstrated only in participants in the affected dominant hand group may reflect reliance and motivation to use the dominant hand during daily activities for this group [48,49]. This hypothesis is consistent with Lang et al.’s findings that, when the dominant limb was the paretic limb, daily arm use was greater, and the paretic limb was used for more hours per day [50]. Our hypothesis on why the affected non-dominant hand group in our study may not have improved significantly at any of the timepoints, for any of the kinematic variables, may be attributed to a diminished need and therefore drive to use that hand, since they could somewhat successfully manage activities of daily living with their unaffected dominant hand [26]. The lack of substantial improvements in kinematics demonstrated by the non-dominant hand affected group may also suggest that learned non-use [21,51] of the affected non-dominant hand may have occurred, limiting further recovery of the non-dominant hand, including movement quality and dexterity. These hypotheses will require further testing in a cohort wearing sensors monitoring upper extremity use patterns over an extended period for definitive evaluation.

The small size of the objects utilized in our study may have influenced the findings. In daily life, people rarely reach for small objects, such as the ones used in this study, with their non-dominant hand. Rather, the non-dominant hand is often used to hold or stabilize larger items, particularly when the object’s size requires the use of both hands [52]. Therefore, the results of the study may reflect differences in how the task aligns with each hand’s functional role. This interpretation is further supported by evidence from a recent study suggesting that dominance is acquired from life-long practice with the preferred limb, specifically in task practice of trajectory shaping and tool use [53]. Consequently, participants with an affected dominant hand may have more practice with the specific movement required in the study’s task, potentially contributing to the greater kinematic improvements observed in this group. These findings highlight the need to consider assessments that target the specific functional role of the affected hand.

### Limitations

One limitation is the small sample size used in this study, particularly when grouping by handedness. We attempted to mitigate this limitation by using Cook’s distance to identify influential data points that might have skewed the analyses of our small sample and run robust mixed linear models for those variables. While our study examined kinematic measures collected with an optical motion capture system using near-infrared active markers, a technology that has been used for several decades, emerging technologies may offer more flexibility in future rehabilitation studies [54]. Furthermore, while our study looked at handedness, we did not examine hemispheric dominance in our analysis. While numerous studies have investigated hemispheric lateralization and its role in motor recovery [19,20,21,23], their work has primarily been conducted on right-handed individuals where they infer hemispheric dominance rather than directly comparing dominant versus non-dominant hand impairments. While this exploratory study helped to detect trends in the role of dominance in stroke recovery, a larger, prospectively designed four-group study, specifically designed to investigate the differential effects of handedness, hemispheric dominance, lesion location and volume would be necessary to disentangle these factors.

## 5. Conclusions

In the primary analysis, subjects showed rapid improvements during the early phase of recovery in both clinical scores and kinematic variables, with the kinematic measures plateauing before the clinical measures, except for hand trajectory smoothness, which improved significantly between day 60 and day 120. In the secondary exploratory analysis, the affected dominant hand group demonstrated significant early clinical and kinematic recovery. However, high rates of early recovery were observed in the non-dominant affected group only for the clinical scores but not for the kinematic features of reach to grasp. The findings indicate that hand dominance may appear to be associated with different motor recovery trajectories and highlight the consideration that rehabilitation strategies might need to be tailored to the functional role of the affected hand. Larger prospective studies are needed to confirm these findings and further examine the role of hand dominance in recovery post-stroke.

## Figures and Tables

**Table 1:** Participant characteristics.

Participant	Age	Sex	UEFMA	Hemisphere Affected	Handedness	Affected Hand	Type of Stroke
	**Dominant Hand Affected Group**						
1	49	Female	48.31	Left	Right	Right	Ischemic
4	49	Female	21.40	Left	Right	Right	Ischemic
6	61	Male	43.056	Left	Right	Right	Ischemic
7	70	Male	39.87	Left	Right	Right	Ischemic
8	45	Male	31.70	Left	Right	Right	Ischemic
9	68	Male	36.98	Left	Right	Right	Ischemic
10	67	Female	33.00	Left	Right	Right	Ischemic
12	55	Female	50.77	Left	Right	Right	Hemorrhagic
14	47	Male	23.46	Right	Left	Left	Ischemic
17	33	Male	51.70	Right	Left	Left	Ischemic
	**Non-Dominant Hand Affected Group**						
2	45	Male	40.05	Right	Right	Left	Ischemic
3	57	Female	39.97	Left	Left	Right	Ischemic
5	54	Male	37.83	Right	Right	Left	Hemorrhagic
11	67	Male	34.05	Right	Right	Left	Ischemic
13	73	Female	40.88	Right	Right	Left	Ischemic
15	65	Female	17.69	Right	Right	Left	Ischemic
16	60	Male	36.38	Right	Right	Left	Ischemic
18	64	Female	36.90	Right	Right	Left	Hemorrhagic
19	72	Female	24.45	Right	Right	Left	Ischemic
20	80	Male	39.10	Right	Right	Left	Ischemic

Note: UEFMA = Upper Extremity Fugl-Meyer Assessment.

**Table 2:** Piecewise linear mixed-effects model estimates for clinical measures.

Model Parameter	BBT Score Estimate (SE)	ARAT Score Estimate (SE)	UEFMA Score Estimate (SE)
**Fixed Effects**			
Intercept	13.65 (3.53)	23.14 (2.56)	36.38 (1.78)
Days 15–30	0.62 (0.12)	0.79 (0.13)	0.78 (0.09)
Days 30–45	0.54 (0.12)	0.57 (0.13)	0.49 (0.09)
Days 45–60	0.31 (0.12)	0.31 (0.13)	0.20 (0.09)
Days 60–120	0.13 (0.03)	0.05 (0.03)	0.04 (0.02)
Days 120–180	0.05 (0.03)	0.02 (0.03)	0.02 (0.02)
**Random Effects**			
Intercept (SD)	14.45	9.64	6.65
Residual (SD)	5.34	6.12	4.35

Note: Fixed effect values are reported as estimates with standard errors (SEs). Random effect values are reported as standard deviations (SDs). BBT = Box and Block Test; ARAT = Action Research Arm Test; UEFMA = Upper Extremity Fugl-Meyer Assessment.

**Table 3:** Piecewise linear mixed-effects model estimates for kinematic measures.

Model Parameter	Time to Peak Velocity (ms) Estimate (SE)	Time After Peak Velocity (ms) Estimate (SE)	Reaching Trajectory Smoothness Estimate (SE)	Path Linearity Estimate (SE)	Reach Duration (ms) Estimate (SE)	Grasp Duration (ms) Estimate (SE)
**Fixed Effects**						
Intercept	475.7 (35.4)	739.7 (53.4)	3.50 (0.09)	1.98 (0.09)	1213.7 (77.2)	319.4 (37.1)
Days 15–30	−7.2 (2.5)	−11.0 (3.5)	−0.02 (0.005)	−0.01 (0.005)	−17.8 (5.2)	−7.5 (2.7)
Days 30–45	−4.2 (2.5)	−7.4 (3.5)	−0.01 (0.005)	−0.01 (0.005)	−11.4 (5.2)	−5.3 (2.7)
Days 45–60	−1.6 (2.5)	−3.5 (3.5)	−0.004 (0.005)	−0.005 (0.005)	−5.1 (5.2)	−2.5 (2.7)
Days 60–120	−0.9 (0.6)	−1.6 (0.9)	−0.005 (0.001)	−0.001 (0.001)	−2.6 (1.3)	−0.6 (0.7)
Days 120–180	0.01 (0.6)	0.07 (0.9)	−0.0003 (0.001)	0.0002 (0.001)	0.1 (1.3)	−0.1 (0.7)
**Random Effects**						
Intercept (SD)	102.5	170.0	0.3	0.3	242.9	103.7
Residual (SD)	120.5	167.5	0.2	0.2	245.5	129.7

Note: Fixed effect values are reported as estimates with standard errors (SEs). Random effect values are reported as standard deviations (SDs).

**Table 4:** Day 15 differences between groups for clinical and kinematic variables.

Variable	Affected Dominant Hand	Affected Non-Dominant Hand	Test Statistic	*p* Value
Age	54.4 ± 12.0	63.7 ± 10.2	t = 1.87	0.08
BBT Score	13.00 ± 10.49	10.00 ± 10.45	t = −0.507	0.62
ARAT Score (0–57)	25.42 ± 13.11	20.86 ± 12.53	t = −0.795	0.44
UEFMA Score (0–66)	38.42 (29.64, 48.92)	37.37 (31.65, 39.99)	U = 38 z = −0.605	0.58
TTPV (ms)	464.27 (362.70, 615.84)	368.01 (194.02, 566.65)	U = 33 z = −1.285	0.22
TAPV (ms)	775.69 ± 388.39	703.78 ± 415.42	t = −0.400	0.70
RTS	3.46 (3.455, 3.87)	3.18 (3.01, 4.27)	U = 39 z = −0.832	0.44
Path Linearity	1.86 ± 0.40	2.11 ± 0.75	t = 0.919	0.37
Reach Duration (ms)	1206.35 (852.10, 1478.94)	850.13 (684.46, 1535.15)	U = 36 z = −1.058	0.32
Grasp Duration (ms)	261.49 (170.03, 384.65)	140.44 (87.33, 507.53)	U = 34 z = −1.209	0.25

Note: Mean, standard deviations, and t values for each variable for each group. Median, interquartile range values, as well as U and z values are provided for non-parametric variables (UEFMA, TTPV, RTS, Reach Duration, and Grasp Duration). BBT = Box and Block Test; ARAT = Action Research Arm Test; UEFMA = Upper Extremity Fugl-Meyer Assessment; TTPV = Time to Peak Velocity; RTS = Reaching Trajectory Smoothness; TAPV = Time After Peak Velocity.

**Table 5:** Piecewise linear mixed-effects model estimates for clinical measures by hand dominance.

Model Parameter	BBT Score Estimate (SE)		ARAT Score Estimate (SE)		UEFMA Score Estimate (SE)	
	Dominant Hand	Non-Dominant Hand	Dominant Hand	Non-Dominant Hand	Dominant Hand	Non-Dominant Hand
**Fixed Effects**						
Intercept	17.07 (4.43)	10.63 (4.33)	25.42 (3.10)	20.86 (3.98)	38.02 (2.45)	34.73 (2.54)
Days 15–30	0.73 (0.18)	0.54 (0.15)	0.93 (0.19)	0.66 (0.17)	0.85 (0.12)	0.72 (0.13)
Days 30–45	0.65 (0.18)	0.45 (0.15)	0.59 (0.19)	0.54 (0.17)	0.51 (0.12)	0.47 (0.13)
Days 45–60	0.26 (0.18)	0.35 (0.15)	0.26 (0.19)	0.37 (0.17)	0.15 (0.12)	0.26 (0.13)
Days 60–120	0.11 (0.05)	0.15 (0.04)	0.02 (0.05)	0.08 (0.04)	0.02 (0.03)	0.07 (0.03)
Days 120–180	0.04 (0.05)	0.06 (0.04)	−0.01 (0.05)	0.06 (0.04)	0.01 (0.03)	0.02 (0.03)
**Random** **Effects**						
Intercept (SD)	12.24	12.67	7.36	11.23	6.60	44.29
Residual (SD)	6.04	5.20	6.56	5.66	4.08	20.29

Note: Fixed effect values are reported as estimates with standard errors (SEs). Random effect values are reported as standard deviations (SDs). BBT = Box and Block Test; ARAT = Action Research Arm Test; UEFMA = Upper Extremity Fugl-Meyer Assessment.

**Table 6:** Piecewise linear mixed-effects model estimates for kinematic measures by hand dominance.

**Model** **Parameter**	**Time to Peak Velocity (ms)** **Estimate (SE)**		**Time After Peak Velocity (ms)** **Estimate (SE)**		**Reaching Trajectory** **Smoothness** **Estimate (SE)**	
	**Dominant Hand**	**Non-Dominant Hand**	**Dominant Hand**	**Non-Dominant Hand**	**Dominant Hand**	**Non-Dominant Hand**
**Fixed Effects**						
Intercept	483.3 (31.1)	467.3 (65.3)	775.69 (73.38)	703.78 (78.71)	3.50 (0.10)	3.51 (0.14)
Days 15–30	−6.3 (1.8)	−7.8 (4.8)	−15.08 (4.45)	−6.86 (5.38)	−0.02 (0.004)	−0.014 (0.009)
Days 30–45	−4.1 (1.8)	−4.5 (4.8)	−10.31 (4.45)	−4.49 (5.38)	−0.01 (0.004)	−0.009 (0.009)
Days 45–60	−0.9 (1.8)	−2.2 (4.8)	−3.63 (4.45)	−3.42 (5.38)	−0.002 (0.004)	−0.006 (0.009)
Days 60–120	−1.2 (0.5)	−0.7 (1.2)	−0.38 (1.11)	−2.78 (1.35)	−0.004 (0.001)	−0.005 (0.002)
Days 120–180	−0.1 (0.5)	0.1 (1.2)	0.33 (1.11)	−0.18 (1.35)	0.0003 (0.001)	−0.0008 (0.002)
**Random Effects**						
Intercept (SD)	74.9	128.6	177.8	171.5	0.29	0.33
Residual (SD)	59.7	161.4	149.2	180.4	0.15	0.30
**Variables Cont.**	**Path Linearity** **Estimate (SE)**		**Reach Duration (ms)** **Estimate (SE)**		**Grasp Duration (ms)** **Estimate (SE)**	
	**Dominant Hand**	**Non-Dominant Hand**	**Dominant Hand**	**Non-Dominant Hand**	**Dominant Hand**	**Non-Dominant Hand**
**Fixed Effects**						
Intercept	1.9 (0.1)	2.1 (0.1)	1260.3 (93.0)	1167.2 (127.3)	269.7 (17.8)	274.8 (52.0)
Days 15–30	−0.01 (0.005)	−0.02 (0.009)	−21.7 (5.2)	−14.0 (9.1)	−8.7 (1.5)	−3.8 (3.2)
Days 30–45	−0.01 (0.005)	−0.01 (0.009)	−14.2 (5.2)	−8.5 (9.1)	−4.7 (1.5)	−3.1 (3.2)
Days 45–60	−0.003 (0.005)	−0.006 (0.009)	−4.6 (5.2)	−5.6 (9.1)	−1.8 (1.5)	−2.2 (3.2)
Days 60–120	0.00003 (0.001)	−0.003 (0.002)	−1.6 (1.3)	−3.7 (2.3)	−0.02 (0.4)	−1.2 (0.8)
Days 120–180	0.0005 (0.001)	−0.00006 (0.002)	0.2 (1.3)	−0.04 (2.3)	0.01 (0.4)	−0.2 (0.8)
**Random Effects**						
Intercept (SD)	0.2	0.4	238.0	261.6	22.5	123.4
Residual (SD)	0.2	0.3	172.6	306.1	50.1	108.5

Note: Fixed effect values are reported as estimates with standard errors (SEs). Random effect values are reported as standard deviations (SDs).

## Data Availability

The data presented in this study are not publicly available due to privacy of the participants but are available from the corresponding author on reasonable request.

## Abbreviations

The following abbreviations are used in this manuscript:

UE	Upper Extremity
BBT	Box and Block Test
UEFMA	Upper Extremity Fugl-Meyer Assessment
ARAT	Action Research Arm Test
RTG	Reach to Grasp
PV	Peak Velocity
TTPV	Time to Peak Velocity
TAPV	Time After Peak Velocity
RTS	Reaching Trajectory Smoothness
